# A Comparative Study of Different Traditional and Bioactive Indirect Pulp Lining Materials on Post-Operative Dentin Hypersensitivity: An In Vivo Study

**DOI:** 10.7759/cureus.76984

**Published:** 2025-01-06

**Authors:** Ahmed A Abdelaziz, Mai S Elgohary, Mohamed A Atiya, Hebatallah A Saleh

**Affiliations:** 1 Department of Conservative Dentistry, Faculty of Oral and Dental Medicine, Badr University in Cairo, Cairo, EGY; 2 Department of Prosthodontics, Faculty of Oral and Dental Medicine, Badr University in Cairo, Cairo, EGY; 3 Department of Dentistry, Ministry of Health Holdings, Cairo, EGY; 4 Department of Conservative Dentistry, Faculty of Dentistry, Cairo University, Cairo, EGY

**Keywords:** bioactive bonding agent, indirect pulp lining, post-operative hypersensitivity, theracal lc, universal adhesive

## Abstract

Aim

The aim of the study was to compare different traditional and bioactive indirect pulp lining materials on post-operative dentin hypersensitivity (POH).

Materials and methods

A total of 72 patients were selected for this study based on specific inclusion and exclusion criteria. They were divided into four main groups, 18 patients each, according to pulp lining material placed (calcium hydroxide, TheraCal LC, universal bonding, or bioactive bonding according to manufacturers’ instructions) and then subdivided into two subgroups according to restorative material used (resin composite or glass ionomer). Patients were evaluated clinically for POH and radiographically for the presence of any preapical lesion at different time periods: immediately after the procedure, at one week, at one month, at three months, and at six months. A verbal analog scale was used for pain during the POH assessment. The response of the patient verbally was recorded for pain scoring and graded from 0 to 4, where 0 means no pain and 4 means intensifying or unbearable pain. The data were calculated, and data analysis was performed using a multiple-way ANOVA test, where p-values of ≤0.05 were considered significant.

Results

There was no significant difference between resin composite and glass ionomer restorations in all groups at different time periods. Calcium hydroxide and universal bond restored with resin composite recorded the highest scores of POH at three and six months than other groups. No preapical lesions were observed in all groups at different time periods.

Conclusions

It can be concluded that using different materials as indirect pulp lining in deep cavities may promote POH. TheraCal LC and bioactive adhesives are considered promising materials for Indirect pulp lining in deep cavities.

Clinical recommendation

Follow-up of patients should be undertaken to recognize any pain that may develop after indirect pulp capping, determine its cause, and treat it early.

## Introduction

Dental caries was and still is one of the most common oral problems in the world. Long-standing carious lesions can progress to involve pulpal tissue, lead to pain and infection, and compromise the quality of life [1]. Management of deep carious and grossly decayed teeth is often challenging. The main objective of the management of deep carious lesions is to preserve the pulp vitality of the teeth with the least trauma as much as possible to the pulp; this helps in providing protection to masticatory forces compared with root canal-treated teeth [2].

Many researchers and clinicians are interested in the management of deep carious lesions. Indirect pulp lining is a therapy protocol where a biocompatible pulp lining material is applied indirectly to the pulp, followed by a restoration that creates a coronal seal to inhibit disease progression and aids in the healing and repair process [3].

The purpose of indirect pulp lining treatment is to save the pulp from microorganisms, as well as physical, chemical, thermal, and electric irritation. It aims to maintain the pulp’s vitality, block the dentinal tubules, and encourage the stimulation of pulp cells and odontoblasts to form dentinal bridges [4]. One or more coatings of pulp lining agent must be applied for the pulp lining treatment. When the remaining dentin thickness is >1 mm, base materials are used under the restorative material, while liners are suggested when the remaining dentin thickness is 0.5-1 mm. Since pulp lining agents come into close contact to the pulp tissue frequently, they should be biocompatible and nontoxic, provide antibacterial properties, offer an ideal seal, reduce microleakage, release fluoride, induce the production of dentinal bridges, and have low toxicity [5]. The materials that can be used for this purpose are varnishes, calcium hydroxide-based products, glass ionomer cement (GIC), and adhesive systems.

The exact mechanism of dentin hypersensitivity is still being researched. The most accepted theory is the hydrodynamic theory. According to this theory, changes in the flow of the fluid in the dentinal tubules can stimulate pain receptors present on nerve endings in the pulp to trigger nerve impulses causing pain [6]. This dentinal fluid flow can be initiated by changes in temperature, humidity, air pressure, and osmotic pressure. Hot and cold food and beverages and physical pressure are typical triggers of dentin hypersensitivity [7].

Hence, the aim of this study was to compare and evaluate the effect of different pulp lining materials, which are calcium hydroxide, TheraCal LC, universal bonding, and bioactive bonding, on post-operative dentinal hypersensitivity. The null hypotheses assumed that there would be no statistically significant difference regarding different pulp lining materials on post-operative hypersensitivity (POH).

The null hypothesis stated that there would be no statistically significant difference between different pulp lining materials on POH.

## Materials and methods

Ethical Committee consent

The current study was granted approval from Badr University in Cairo Institutional Ethical Committee with approval number BUC-IACUC-24-623-102. In addition, the study protocol was also submitted to the US National Institute of Health protocol registry (ClinicalTrials.gov NCT06707311).

Sample size calculation

Power and sample size calculations for a two-proportion test were performed. Specifically, it is testing the null hypothesis that the comparison proportion (p) is equal to the baseline proportion of 0.73, against the alternative hypothesis that they are not equal. The significance level (α) is set to 0.05, which is a commonly used value in hypothesis testing.

As shown in the Results section, for a comparison proportion of 0.01 and a target power of 0.8, the required sample size for each group is 6. The actual power achieved with this sample size is 0.825102, which slightly exceeds the target power.

Additionally, a power curve is provided, which visualizes the relationship between the sample size and the power of the test for different values of the comparison proportion.

This type of analysis is useful when designing studies or experiments to ensure that the sample size is adequate to detect the desired effect size with sufficient statistical power while also controlling the type I error rate (significance level).

Study design

This study was conducted for a six-month duration period and included 72 patients whose posterior teeth molars or premolars with deep class II carious lesions indicated indirect pulp lining. Patients were chosen from the outpatient clinic of the Department of Conservative Dentistry, Faculty of Dentistry, Badr University, Cairo, Egypt. Patients were chosen based on inclusion criteria, including being in good general health, age 20 to 40 years, possessing a vital molar or premolar tooth confirmed through vitality testing (thermal or electrical pulp tester), having class II cavities, deep carious lesions, and the absence of clinical signs or symptoms indicative of a non-vital tooth, such as spontaneous pain, tenderness to percussion, abscess, fistula, periodontal tissue swelling, or abnormal tooth mobility. There must be adequate tooth structure for restoration. Patients exhibited cooperation and motivation.

The exclusion criteria included patients suffering from systemic disorders, including uncontrolled diabetes and cardiovascular conditions, or those who had undergone chemotherapy or radiotherapy; those with a history of spontaneous, unprovoked odontalgia or dental movement (grades I, II, and III); those with sensitivity to percussion, external or internal root resorption, periapical lesions, or the existence of fistulae; and pregnant females. History of drug misuse and previously repaired teeth was excluded to rule out any pulp responses from prior restorations.

Grouping of patients and cavity preparation

**Figure 1:**
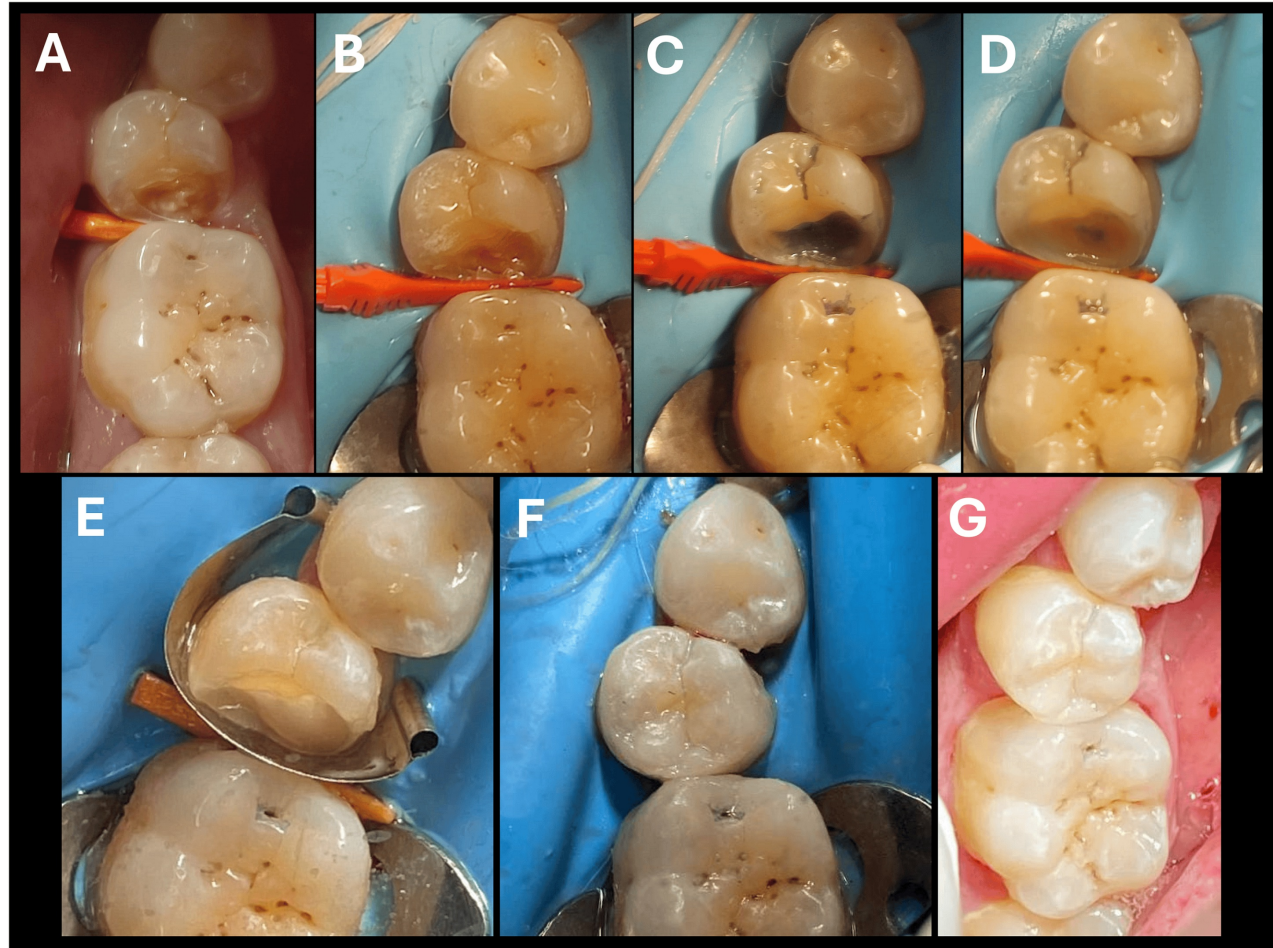
Steps of applying universal bond on dentin as indirect lining material Original photos taken during the study A. Preoperative and pre-wedging for marginal analysis to preserve the gingival tissues. B. Rubber dam isolation. C. Application of caries-detecting dye using Cario Finder. D. Application of All-Bond Universal as a lining material. E. Application of the first layer of 1-mm resin composite over the light-cured bonding agents. F. Complete restoration with resin composite. G. Finishing and polishing.

Upon completion of a comprehensive history questionnaire and the signing of the patient consent form, an examination of both hard and soft tissues was conducted, and the procedures were thoroughly elucidated to the patients. A preoperative periapical radiography was performed to assess the pulpal and periodontal status in accordance with the American Dental Association guidelines. The teeth requiring treatment were subjected to local anesthesia using a single block injection of mepacrine-L, which comprises 20 mg of mepivacaine hydrochloride and 0.06 mg of levonordefrin hydrochloride. Prewedging for marginal analysis to preserve gingival tissues was performed (Figure 1A). A rubber dam was employed to isolate the specified tooth (Figure 1B). The cavity outline was prepared, and all unsupported enamel was mechanically excised using a sterile high-speed round bur with coolant, followed by manual removal with a sharp sterile spoon excavator to eliminate soft carious dentin; all carious dentin was completely removed. The cavity was irrigated with sodium hypochlorite to reduce the bacterial load. The removal of caries was performed until the dentin resisted hand excavation with the sharp spoon excavator. Subsequently, a caries detector dye (Cario Finder) was employed to verify the complete removal of caries and the attainment of sound-affected dentin (Figure 1C). Thereafter, a saddle sectional matrix and appropriately sized wooden wedge were positioned prior to restoration.

This clinical investigation used four distinct indirect pulp lining materials: calcium hydroxide, TheraCal LC, a universal bonding agent, and a bioactive bonding agent. Materials used in the study are shown in Table 1. Patients were categorized into four primary groups based on the type of lining material used beneath the repair, with each group further separated into two subgroups according to the final restorative material employed to restore the prepared cavities as follows: group 1 (G1) patients received calcium hydroxide and restored with resin composite, group 2 (G2) patients received TheraCal LC and restored with resin composite, group 3 (G3) patients received universal bonding and restored with resin composite, group 4 (G4) patients received bioactive bonding and restored with resin composite, group 5 (G5) patients received calcium hydroxide and were restored with glass ionomer, group 6 (G6) patients received TheraCal LC and were restored with glass ionomer, group 7 (G7) patients received bioactive bonding and restored with glass ionomer, and group 8 (G8) patients received bioactive bonding and restored with glass ionomer. Variables used in the study are shown in Table 2.

**Table 1:** Materials used in the study 10-MDP, 10-methacryloyloxydecyl dihydrogen phosphate; 4-AET, 4-acryloxyethyltrimellitic acid; 4-AETA, 4-acryloxyethyltrimellitate anhydride; Bis-GMA, bisphenol A-glycidyl methacrylate; BPDM, biphenyl dimethacrylate; HEA, hydroxyethyl acrylate; HEMA, 2-hydroxyethylmethacylate; TEGDMA, triethylene glycol dimethacrylate; UDMA, urethane dimethacrylate

Material	Manufacturer	Composition and description
Calcium hydroxide (Dycal®)	Dentsply, Milford, DE, USA	Base paste: 1,3-butylene glycol salicylate, calcium phosphate, calcium tungstate, iron oxide pigments. Catalyst paste: calcium hydroxide, n-ethyl-o/p-toluene sulfonamide, zinc oxide, titanium dioxide, zinc stearate, iron oxide pigments.
TheraCal™ LC	BISCO, Schaumburg, IL, USA	10-MDP, BPDM, Bis-GMA, HEA, water, ethanol, photoinitiator. Light-cured, resin-modified calcium silicate material consists of tricalcium silicate particles in a hydrophilic monomer that stimulates hydroxyapatite and secondary dentin bridge formation through calcium release and an alkaline pH.
All-Bond Universal	BISCO, Schaumburg, IL, USA	Universal, light-cured, single-component, ethanol/water-based dental adhesive that combines etching, priming, and bonding in one bottle, bonds to dentin and cut and uncut enamel.
FL-Bond II	Shofu, Kyoto, Japan	Primer: 4-AET, 4AETA, HEMA ethanol, water. Adhesive: 4-AET, UDMA, HEMA, TEGDMA. It is a two-step self-etching fluoride-releasing adhesive system. The FL-Bond II Primer contains a new effective adhesive-promoting monomer and a new photo initiator. The FL-Bond II bonding agent incorporates fluoride-containing S-PRG fillers (surface pre-reacted glass ionomer), which offer a permanently available protection against secondary caries by an optimized remineralization of the adjacent hard tooth structure.
Ventura Nanolux, A2	MADESPA S.A., Toledo, Spain	Universal light-curing nano-hybrid fluoride release composite, modified with nanoceramic particles, filler content 81%, particle size from 0.05 to 0.9 microns.
Nova Glass-F	IMICRYL Dental LLC, Konya, Turkey	Radiopaque glass ionomer filling cement

**Table 2:** Variables used in the study

Group	Subgroup (restoration material used)	Time				
		Immediate	After one week	After one month	After three months	After six months
Calcium hydroxide	Resin composite	G1	G1	G1	G1	G1
	Glass ionomer	G5	G5	G5	G5	G5
TheraCal LC	Resin composite	G2	G2	G2	G2	G2
	Glass ionomer	G6	G6	G6	G6	G6
Universal bond	Resin composite	G3	G3	G3	G3	G3
	Glass ionomer	G7	G7	G7	G7	G7
Bioactive bond	Resin composite	G4	G4	G4	G4	G4
	Glass ionomer	G8	G8	G8	G8	G8

In groups G1 and G5, as per the manufacturer's instructions, calcium hydroxide-based liner (Dycal) was applied, cavity preparation was done, and then the cavity was rinsed with 2.6%-5% sodium hypochlorite. Then, the cavity was gently dried with a cotton pellet without overdryness or desiccation. After that, equal volumes of base and catalyst pastes were dispensed on the paper pad provided. Using a Dycal liner applicator, the mix was stirred immediately thoroughly until a uniform color was achieved without over-saturation; mixing was completed within 10 seconds. Using the ball-pointed Dycal liner applicator or similar instrument, a thin layer of the mix, approximately 0.8-1 mm, was placed directly on the deepest part of the cavity in dentin, which was judged to be less than 1 mm remaining dentin bridge thickness, avoiding placing the material on enamel or the margins of the cavity, also placing a large bulk of material was avoided. Finally, the Dycal liner was allowed to be completely set. The mixed material was set for approximately 2-3 minutes on the mixing pad under normal room conditions (70°F with 50% relative humidity). Set time is shorter in the mouth due to moisture and temperature. After complete setting, any set excess was removed from retention areas, enamel, and margins with a sharp spoon excavator [8].

In groups G2 and G6, the cavity was prepared, rinsed, and then dried as previously mentioned, TheraCal LC was applied to the clean, dry cavity in a layer of 1 mm or less directly on the deepest part of dentin and then light cured for 20 seconds according to manufacturer’s instructions using Woodpecker light curing unit.

In groups G3 and G7, after cavity preparation, the cavity was washed thoroughly with water spray, and then selective etching was performed on the enamel surface for 30 seconds and again the cavity was rinsed thoroughly for 20 seconds. Excess water was removed by blotting the surface with an absorbent pellet for 1-2 seconds, leaving the preparation visibly moist. Two separate coats of All-Bond Universal were applied on the dentin surface, scrubbing the preparation with a micro brush for 10-15 seconds per coat. Excess solvent was evaporated by thoroughly air-drying with an air syringe for at least 10 seconds until the surface had a uniform glossy appearance (Figure 1D); otherwise, an additional coat of All-Bond Universal was applied, and steps 2 and 3 were repeated. The adhesive was light-cured for 10 seconds and then the cavity was restored with the restorative material according to the manufacturer’s instructions [9], as shown in Figure 1F. After that, finishing and polishing were accomplished (Figure 1G).

In groups G4 and G8, self-adhesive two-step bioactive FL-Bond II was used. After the cavity was prepared, rinsed, and dried as previously mentioned, primer was applied to the enamel and dentin for 20 seconds and then air dryness for 5 seconds; a single coat of the bonding agent was applied to the enamel and dentin using a micro brush in a scrubbing motion. The adhesive was light-cured for 20 seconds and then the cavity was restored with the restorative material according to the manufacturer's instructions [10].

A verbal analog scale was used for pain scoring during POH assessment. It has the advantage of describing the pain using patient’s own words and helps follow up the stages of pain through the whole period of this study. The verbal response of the patient was recorded on a sheet for pain scoring, ranging from 0 to 4, where 0 means no pain and 4 means intensifying or unbearable pain.

**Figure 2:**
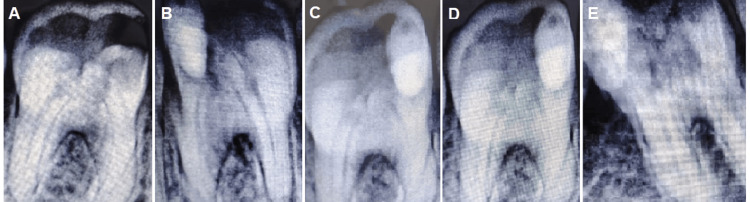
Radiographic evaluation of G4 at different time periods Original radiographic images taken during the study A. Preoperative showing proximal caries in molar tooth. B. Immediate post-operative radiograph after restoration. C. Image after one month showing initiation of reparative dentin formation. D. Image after three months showing increased reparative dentin formation. E. Image after six months showing a well-defined layer of reparative dentin under the restoration.

Patients in all eight groups were assessed clinically and radiographically preoperatively (Figure 2A) and immediately post-procedure (Figure 2B), as well as at one week, one month (Figure 2C), three months (Figure 2D), and six months (Figure 2E), using the following criteria: (1) lack of spontaneous pain and/or sensitivity to percussion; (2) lack of edema, sinus, fistula, or aberrant mobility; (3) lack of radiolucency in the inter-radicular and/or periapical areas; and (4) lack of external or internal root resorption. The data were computed, and analysis was conducted.

Statistical analysis was conducted using a commercially available software application (SPSS Version 20, IBM Corp., Armonk, NY, USA) for Windows.

Numerical data were summarized using mean, standard deviation, confidence intervals, and range. The data were assessed for normalcy by examining the distribution and using the Kolmogorov-Smirnov and Shapiro-Wilk tests. Groups were compared using the Kruskal-Wallis test based on the non-parametric distribution of data. The Mann-Whitney U test was employed for comparisons between the two groupings. The Friedman test and Wilcoxon signed-rank test were employed to examine the impact of time. The multiple ways ANOVA test was employed to examine the interaction of study variables. All p-values were bilateral. P-values equal to or less than 0.05 were deemed significant.

## Results

Comparison between groups

**Figure 3:**
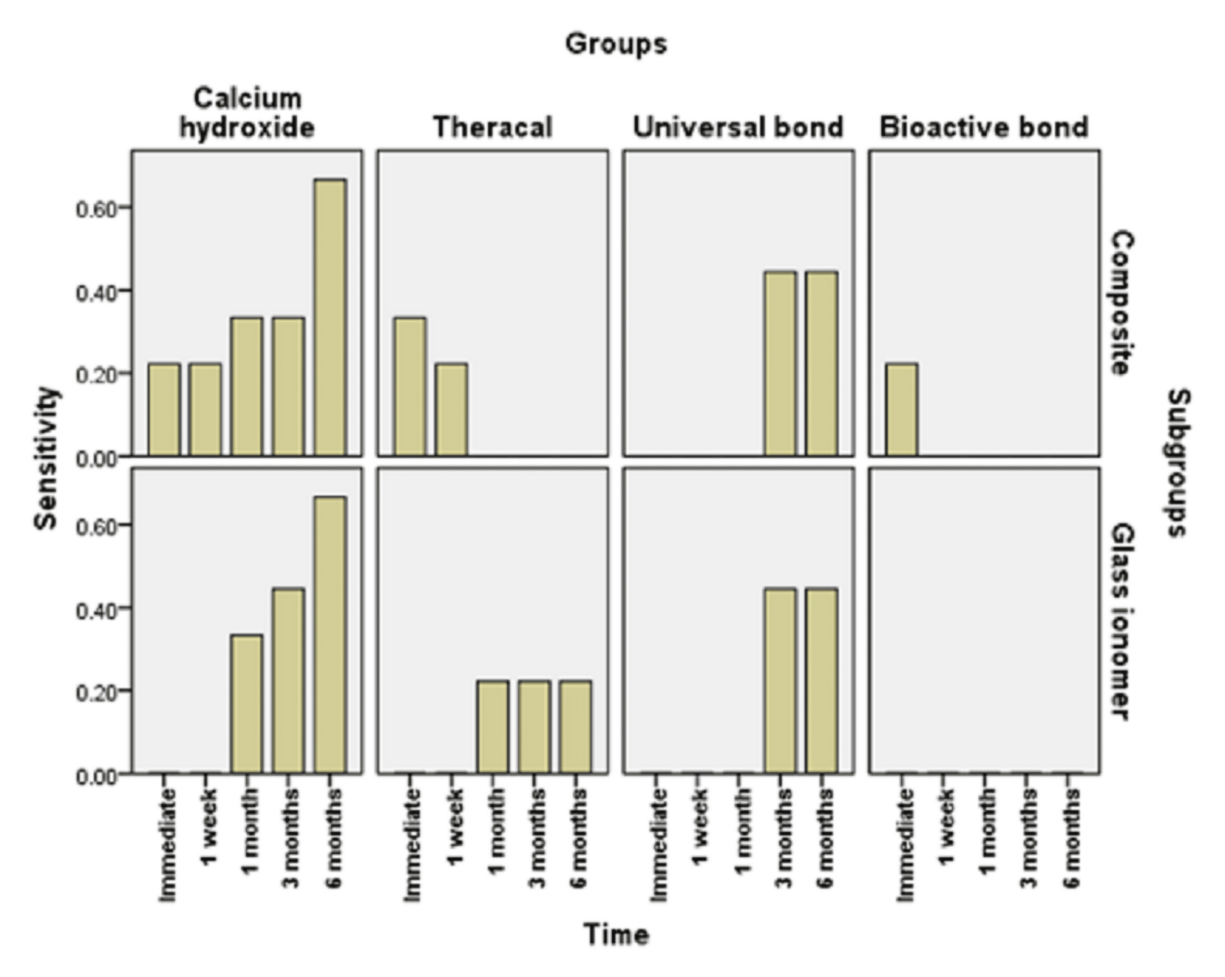
Bar chart illustrating the mean value of sensitivity score in different groups

**Figure 4:**
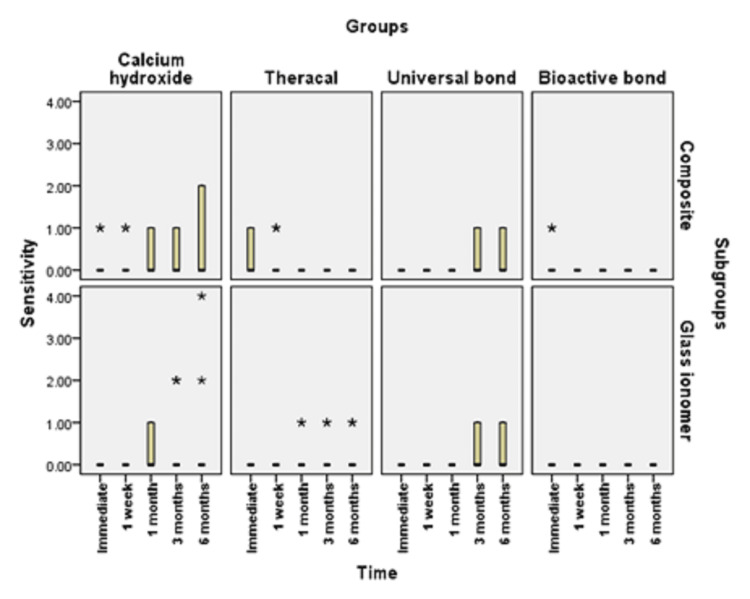
Bar chart illustrating the median value of sensitivity score in different groups In a boxplot, outliers are denoted with an asterisk. The bottom whisker extends to the lowest value that is not an outlier and the upper whisker extends to the highest value that is not an outlier. Extreme outliers are data points that are more extreme than Q1 - 3 x IQR or Q3 + 3 x IQR. Extreme outliers are marked with an asterisk (*) on the boxplot. Mild outliers are data points that are more extreme than Q1 - 1.5 x IQR or Q3 + 1.5 x IQR but are not extreme outliers. Mild outliers are marked with a circle (O) on the boxplot.

Figures 3, 4 show the comparison between different groups. Immediate scoring of POH in groups restored with resin composite showed that calcium hydroxide and bioactive bond recorded a mean score of 0.22±0.44, median 0, TheraCal LC recorded a mean score of 0.33±0.50, median 0, and universal bond recorded a mean score of 0.00±0.00, median 0. The difference between groups was not statistically significant (p=0.351). For the glass ionomer, all groups recorded a mean score of 0.00±0.00 (median of 0), with no difference between groups (p=1).

After one week, the scoring of POH in groups restored with resin composite showed that calcium hydroxide and TheraCal LC recorded a mean score of 0.22±0.44, median 0, while universal bond and bioactive bond recorded a mean score of 0.00±0.00, median 0. The difference between groups was not statistically significant (p=0.224). With glass ionomer, all groups recorded a mean score of 0.00±0.00, median 0, with no difference between groups (p=1).

After one month, groups restored with resin composite showed that calcium hydroxide recorded a mean score of 0.33±0.50, median 0. This value was significantly greater than each of TheraCal LC, universal bond, and bioactive bond, which recorded a mean score of 0.00±0.00, median 0 (p=0.023). With glass ionomer, calcium hydroxide recorded a mean score of 0.33±0.50, median 0, while TheraCal LC recorded a mean score of 0.22±0.44, median 0. Universal bond and bioactive bond each recorded a mean score of 0.00±0.00, median 0. The difference between groups was not statistically significant (p=0.107).

At the three-month assessment of POH, groups restored with resin composite showed that calcium hydroxide recorded a mean score of 0.33±0.50, median 0, while universal bond recorded a mean of 0.44±0.53, median 0. Values recorded in these groups were significantly greater than each of TheraCal LC and bioactive bond, which recorded a mean score of 0.00±0.00, median 0 (p=0.032). For those restored with glass ionomer, calcium hydroxide recorded a mean score of 0.44±0.88, median 0. TheraCal LC recorded a mean score of 0.22±0.44, median 0, universal bond recorded a mean score of 0.44±0.53, median of 0, and bioactive bond recorded 0.00±0.00, median 0. The difference between groups was not statistically significant (p=0.212).

At the six-month assessment of POH, groups restored with resin composite showed that calcium hydroxide recorded a mean score of 0.67±1, median 0, while universal bond recorded a mean of 0.44±0.53, median 0. Values recorded in these groups were significantly greater than each of TheraCal LC and bioactive bond, which recorded a mean score of 0.00±0.00, median 0 (p=0.039). For those restored with glass ionomer, calcium hydroxide recorded a mean score of 0.67±1.41, median 0. TheraCal LC recorded a mean score of 0.22±0.44, median 0, universal bond recorded a mean score of 0.44±0.53, median 0, and bioactive bond recorded a mean score of 0.00±0.00, median 0. The difference between groups was not statistically significant (p=0.212).

Comparison between subgroups

**Figure 5:**
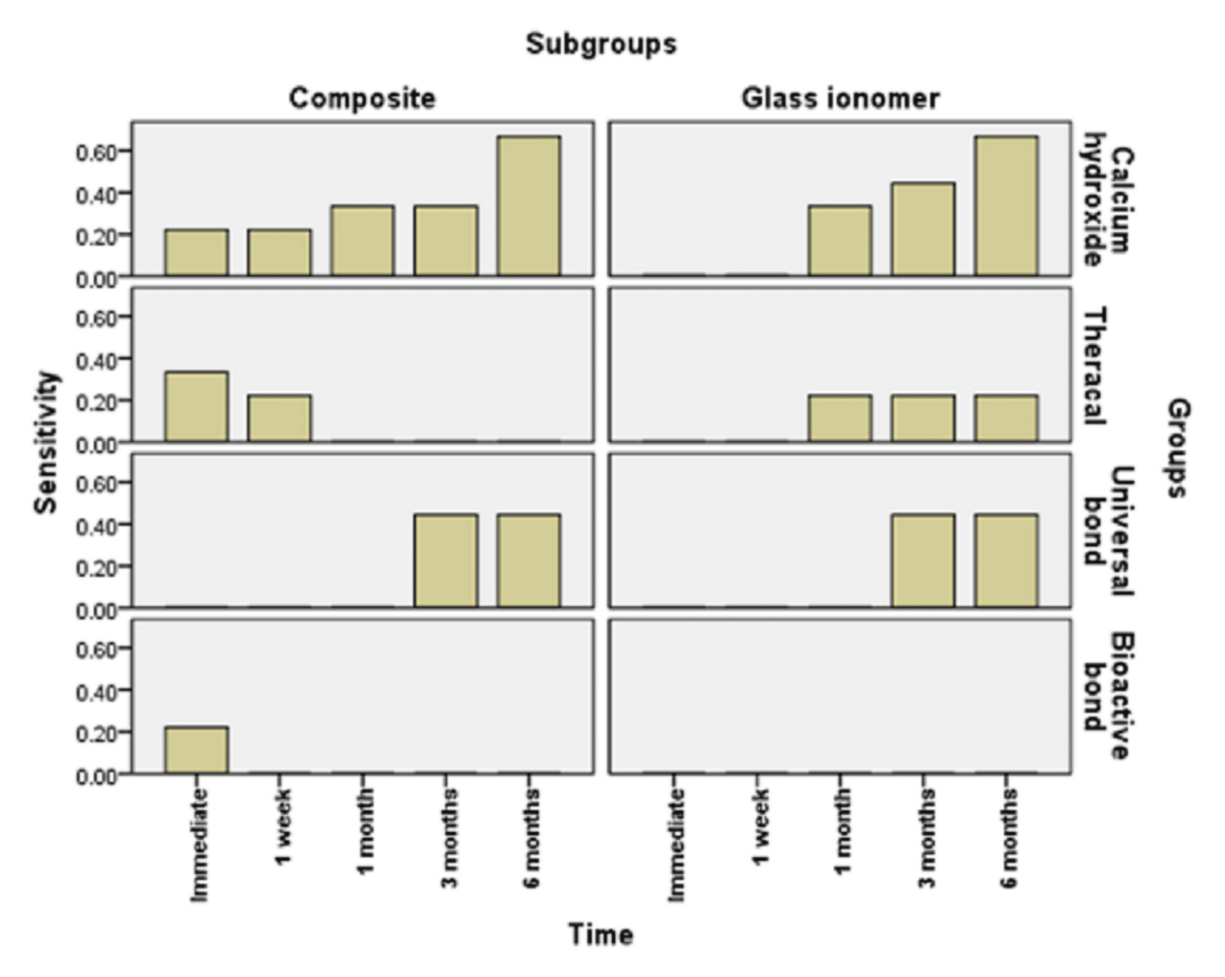
Bar chart illustrating the mean value of sensitivity score in subgroups

**Figure 6:**
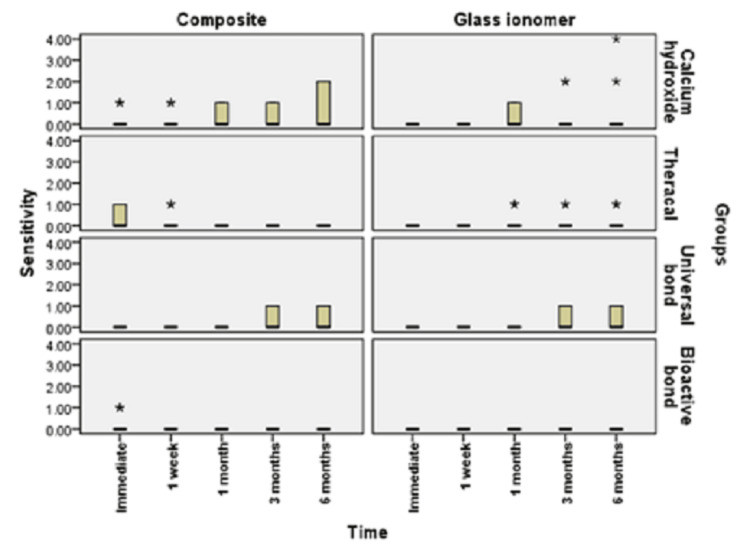
Bar chart illustrating the median value of sensitivity score in subgroups In a boxplot, outliers are denoted with an asterisk. The bottom whisker extends to the lowest value that is not an outlier and the upper whisker extends to the highest value that is not an outlier. Extreme outliers are data points that are more extreme than Q1 - 3 x IQR or Q3 + 3 x IQR. Extreme outliers are marked with an asterisk (*) on the boxplot. Mild outliers are data points that are more extreme than Q1 - 1.5 x IQR or Q3 + 1.5 x IQR, but are not extreme outliers. Mild outliers are marked with a circle (O) on the boxplot.

Results summarized in Figures 5, 6 showed that for calcium hydroxide, there was no significant difference between resin composite and glass ionomer subgroups immediately (p=0.145), after one week (p=0.145), after one month (p=1), after three months (p=0.866), and after six months (p=0.735). For TheraCal LC, there was no significant difference between resin composite and glass ionomer subgroups immediately (p=0.065), after one week (p=0.145), after one month (p=0.145), after three months (p=0.145), and after six months (p=0.145). Also, for universal bond, there was no significant difference between resin composite and glass ionomer subgroups immediately (p=1), after one week (p=1), after one month (p=1), after three months (p=1), and after six months (p=1). Finally, for bioactive bond, there was no significant difference between resin composite and glass ionomer subgroups immediately (p=0.145), after one week (p=1), after one month (p=1), after three months (p=1), and after six months (p=1).

Effect of time

**Figure 7:**
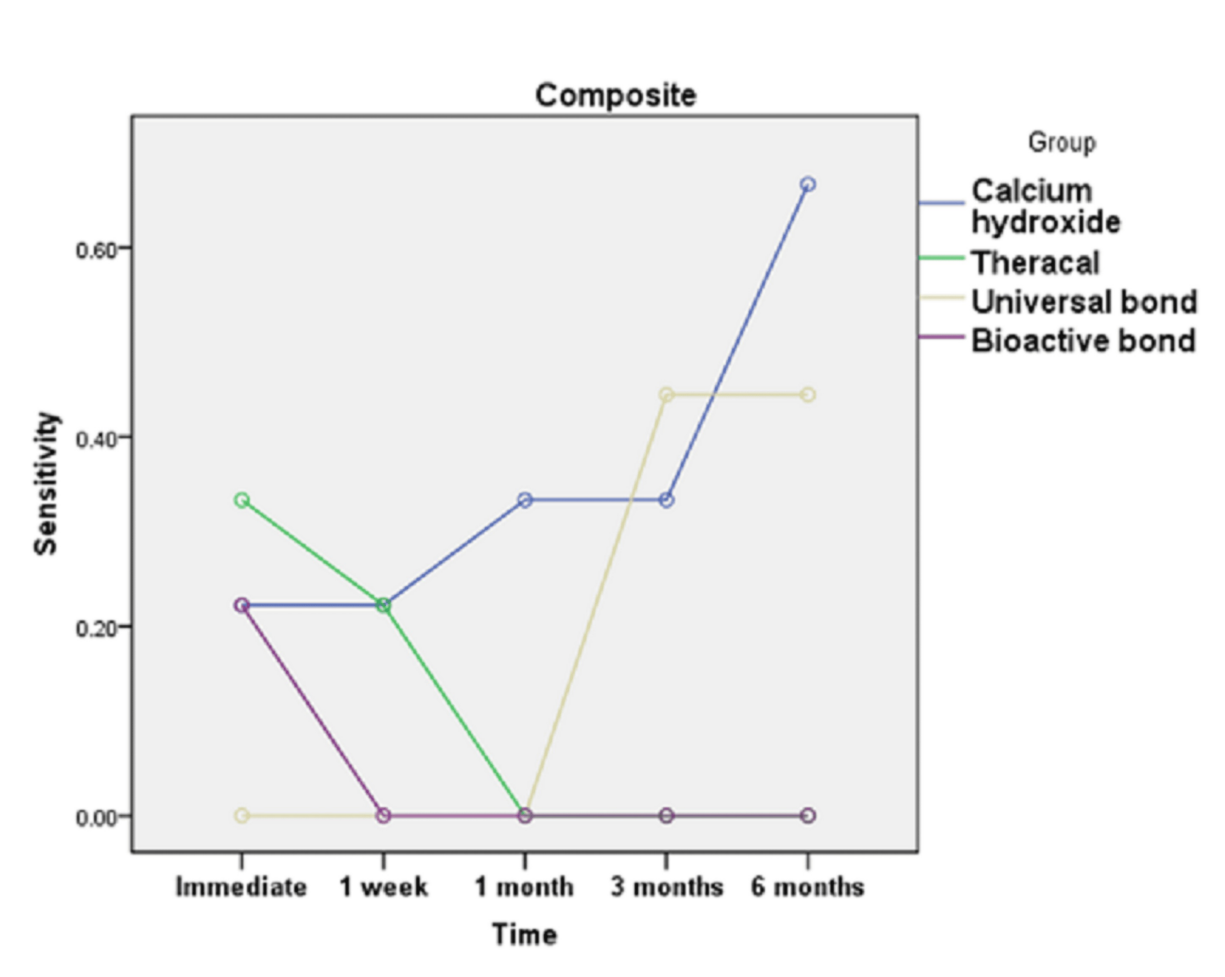
Line chart illustrating the effect of time on POH score in different groups restored with resin composite POH, post-operative dentin hypersensitivity

**Figure 8:**
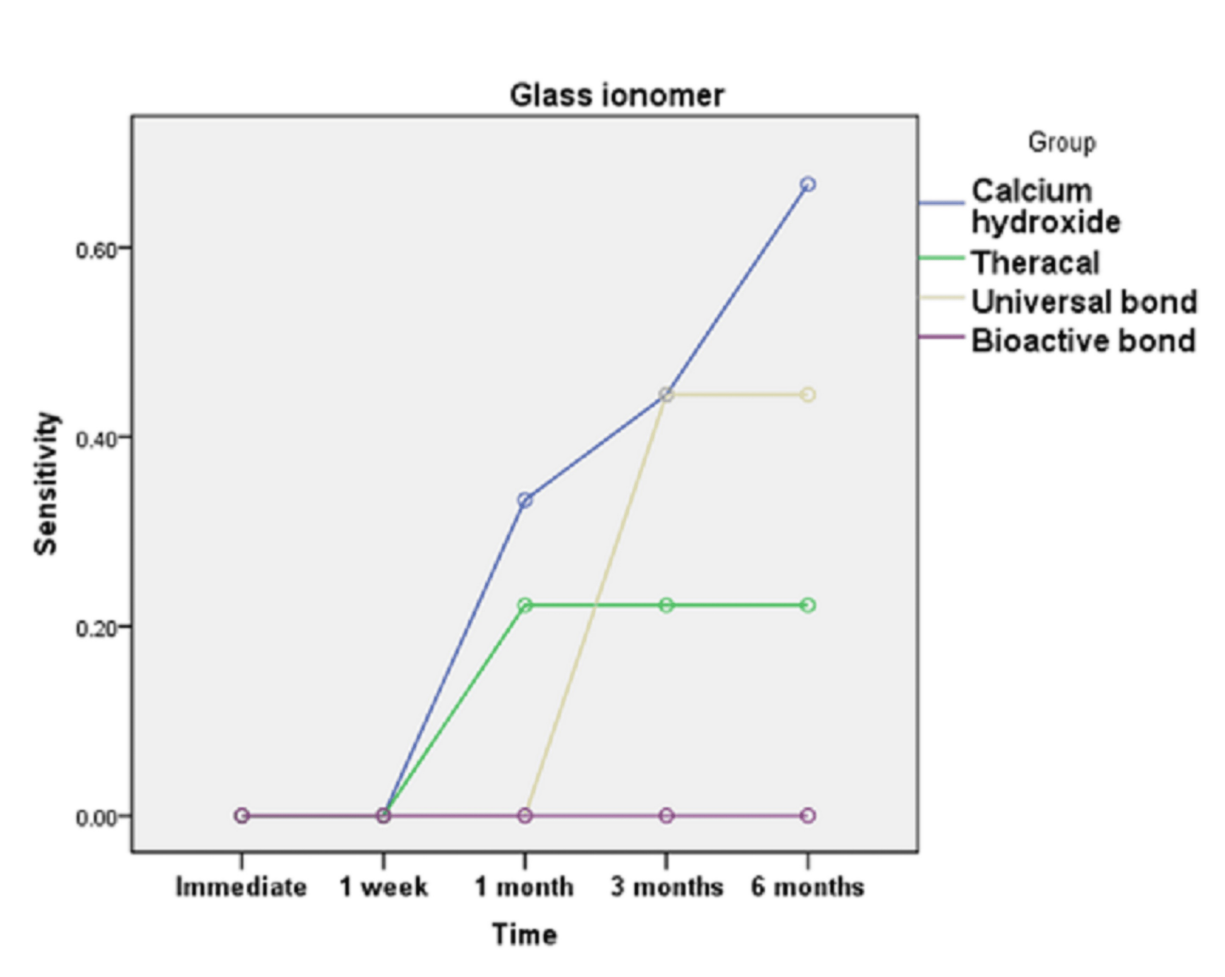
Line chart illustrating the effect of time on POH score in different groups restored with glass ionomer POH, post-operative dentin hypersensitivity

Figures 7, 8 showed that for calcium hydroxide restored with resin composite, the value recorded for POH at six months was significantly greater than the value recorded in the preceding time periods (p=0.035). Post hoc test revealed no significant difference between immediate, one week, one month, and three months. For universal bond restored with resin composite, the value recorded for POH at three and six months was significantly greater than the value recorded in the preceding time periods (p=0.003). Finally, TheraCal LC restored with resin composite and bioactive bond restored with resin composite revealed no statistically significant difference by time (p=0.056 and p=0.092, respectively). On the other hand, calcium hydroxide, TheraCal LC, and bioactive bond restored with glass ionomer revealed no statistically significant difference by time (p=0.133, p=0.092, and p=1, respectively). However, universal bond restored with glass ionomer recorded POH values at three and six months being significantly greater than the values recorded in the preceding time periods (p=0.003).

Interaction of variables

Multiple ways ANOVA revealed that the group variable and the time variable had a statistically significant effect (p=0.000, p=0.002), whereas the group (restored with resin composite and glass ionomer) variable had no significant effect (p=0.621). Moreover, the interaction of the group and time variable had a significant effect (p=0.006).

The power of the study based on group and time variables was 0.989 (99%) and 0.934 (93%), respectively. The results of the interaction of variables are summarized in Table 3.

**Table 3:** Results of multiple ways ANOVA test for the effect of the study variables and their interaction on sensitivity Significance level p≤0.05. *Significant; ns, non-significant.

Source	Type III sum of squares	Df	Mean square	F	p-Value	Partial eta squared	Observed power
Subgroups	0.044	1	0.044	0.245	0.621 ns	0.001	0.078
Group	4.233	3	1.411	7.785	0.000*	0.068	0.989
Time	3.183	4	0.796	4.391	0.002*	0.052	0.934
Subgroups x group	0.111	3	0.037	0.204	0.893 ns	0.002	0.088
Subgroups x time	1.094	4	0.274	1.510	0.199 ns	0.019	0.466
Group x time	5.128	12	0.427	2.358	0.006*	0.081	0.962
Subgroups x group x time	0.861	12	0.072	0.396	0.965 ns	0.015	0.226

## Discussion

This study was designed to evaluate POH in patients after complete caries removal in deep cavities with remaining thin dentin bridge thickness and placement of different materials as pulp lining or lining then complete sealing of the cavity using resin composite and glass ionomer restorations.

The assessment of POH raised from pulp reactions can be made both clinically and radiographically, and hence, in vivo, studies are considered the golden standard that is required to assess pulp response from direct or indirect lining materials [11]. Brännström defined post-operative sensitivity as an unpleasant sensation arising from a pulp reaction to operative procedures; it is caused by polymerization shrinkage and internal stresses that develop between cavity walls and restoration and provoke leakage [12].

Different techniques have been proposed in a trial to eliminate or reduce POH when a remaining dentin bridge thickness is less than 1mm by placing a liner or base to protect the pulp; different materials can be used for such purposes as calcium hydroxide, MTA, GIC, calcium silicate products, and adhesive systems [13].

POH is highly observed with adhesive restorations, especially posterior resin composites; many factors contribute to this problem, such as cavity size and depth, bonding procedures, technique of restoration placement, shrinkage stresses during polymerization, dentin dryness, occlusal discrepancies, micro/nano-leakage due to insufficient sealing of the dentin. Also, this phenomenon might be observed due to the penetration of unreacted or residual monomers of adhesive system or resin composite to the pulp via dentinal tubules through thin dentin bridge thickness, creating hypersensitivity [14,15].

Strict protocols represented by applying Inclusion and Exclusion criteria have been taken into consideration for the selection of patients during this study in order to have a standardized methodology that will decrease bias and not interfere with the final results of this study, also retreatment of previously restored class II was excluded as the pulpal status of such teeth may be altered from previous operative procedures. Patients who met the inclusion criteria and were informed about the procedures and aim of the study signed consent as approval to participate in this research [16].

Dental pulp lining, also referred to as vital pulp therapy, is divided into direct and indirect lining depending on whether there is pinpoint exposure or a remaining thin dentin bridge thickness. Indirect pulp lining is needed to protect the pulp and prevent or minimize post-operative sensitivity, with varying success rates of 73% to 97% reported in many studies. Different indirect pulp lining materials have been used, from which calcium hydroxide remains the golden standard by most clinicians for its power to accelerate the formation of reparative dentin and decrease hypersensitivity. Many questions have been raised about whether it is still necessary to place a lining material in deep cavities under resin composite or whether establishing the perfect seal with the proper application technique of the adhesive system is quite satisfactory with a thin dentin bridge to protect the pulp [17,18].

Different techniques have been evolved for the management of deep carious lesions, including complete, partial, or selective and stepwise caries excavation. In the present study, complete caries excavation has been adopted, where the decayed dentin is completely removed, followed by the application of lining material and final restoration. Nowadays, many studies describe complete caries removal as an overtreatment procedure for the fear of pulp exposure and prefer partial caries removal; others prefer complete caries removal as the progression of the carious lesion is eliminated, clinically most dental practitioners prefer complete caries excavation [19,20].

Calcium hydroxide is still widely used as a pulp lining material in direct and indirect techniques, thanks to its advantage of reparative dentin properties, higher initial PH, antibacterial properties, and precipitation of calcium ions; however, long-term stability against bacterial penetration is questionable due to its gradual degradation over time, presence of tunnel defects and lack of adhesion [21,22].

New indirect pulp lining techniques using dental adhesive systems with precise application may stimulate effective pulp healing without toxic effects and can provide superior results than calcium hydroxide due to the process of dentin hybridization and formation of resin tags due to penetration of bonding agent into dentin and formation of hybrid layer which can protect pulp against bacterial invasion and POH [23,24].

Recently, a universal adhesive system has been introduced into the market. They can be used using total-, self-, or selective etch mode, enabling dentists to select the appropriate application mode for each case. It is applied as one coat or two coats according to manufacturers' instructions and contains nanosized particles for optimal hybridization. In this study, All-Bond Universal was 10-used, besides containing functional monomers MDP, which enhances chelation with dental substrates, facilitating chemical affinity to hydroxyapatite. Although this new adhesive has the ability to decrease the POH and improve the marginal seal, it is questionable whether universal adhesives seal adequately the dentin due to the presence of hydrophilic monomers, which would result in further demineralization and subsequent POH [25,26].

Bioactive self-etching fluoride-releasing bonding agent FL-Bond II was also considered in this study; it is a two-step adhesive system that provides a secure marginal seal with optimal bond strength to enamel and dentin due to the presence of a separate hydrophobic bonding agent. It contains S-PRG fillers that have protection against secondary bacteria with remineralization capabilities [27].

TheraCal LC was used in this study as an indirect pulp lining material; it is a fourth-generation calcium silicate base material with a resin component and can be photopolymerized at a thickness of 1 mm. It has the advantage of the immediate setting, ease of handling, and dispensing besides low solubility, it has high alkalinity with a pH of 10-11 that returns neutral within 3 days, and it has a great capacity for dentin bridge formation and high calcium release capability. It can be used in all deep cavity preparations and has many versatile roles due to its properties once precisely applied [28,29].

GIC was developed by Wilson and Kent in 1971. It gained wide popularity owing to its anticarcinogenic properties due to Fluoride release, chemical adhesion to enamel and dentin-beside biocompatibility, and similar modulus of elasticity to dentin, although it has some limitations such as poor physical properties, high solubility, and dehydration. Nowadays, the incorporation of HEMA in GIC has resulted in enhanced physical and mechanical properties of such material. Until now, it has been observed that placing GIC as a lining or restorative material might reduce the post-operative sensitivity compared to resin composite restorations [30].

In the current research, rubber dam isolation was adopted in all cases (Figure 1B). It is considered the golden technique for moisture control to achieve a successful adhesive restoration. Also, different sizes of wedges were used and dental floss ligatures to improve the seal and visibility of the restorative procedures [31]. Also, all the restorative procedures were placed by an experienced operative dentist following the manufacturers’ instructions for the materials used in the study in order to achieve standardization protocol during the whole procedure.

In the current investigation, a verbal analog scale has been applied for pain scoring during POH assessment; this method has been adopted in previous studies and has the advantage of prescribing the pain by patient real word data and helping to follow up the stages of pain through the whole period on this study. Other studies used a visual analog scale for pain assessment, but it has a limitation in that patients may be positioned on the line of such scale in different places, giving inaccurate readings [32,33].

Assessment of post-operative sensitivity was done immediately, one week, one month, three months, and six months using VAS by application of cold ice as a stimulus using an ice stick and using air stimulus from a three-way air syringe. The response of the patient verbally was recorded on a sheet for pain scoring graduated from 0 to 4, where 0 means no pain and four means intensifying or unbearable pain [34].

In the present assessment, caries detection dye was used to distinguish between infected dentin that must be removed and affected dentin that is preserved as it is capable of remineralization (Figure 1C), and by this method, a thin dentin bridge thickness is maintained for IPC. Also, caries excavation was done using sharp excavators or large round burs running at low speed with light pressure to minimize any pulp reactions that may result from overheating or vibrations [35].

Class II cavity preparation was selected in this study as most clinical studies have reported POH arising in class II resin composite restorations, and this may be attributed to several factors from which are the cuspal deflection, the more destruction of dental substrates and marginal ridges, and the axial depth of the cavity and its proximity to the pulp [36].

Patients' ages in this study ranged from 20-40 years old, with 75% female and 25% male. It was found in previous studies that gender factor has no effect on the POH (POH) [16]. Also, 60% of class II cavity preparation was done on molars and 40% on premolars.

According to the results obtained from this clinical study, it has been observed that the null hypotheses were rejected as there were statistically significant clinical differences among different pulp lining materials on POH. Besides the clinical assessment of the POH, radiographic assessments were conducted using preapical radiographs in all time periods during this study (immediately, after one week, after one month, after three months, and after six months). It was found that using different pulp lining materials with different restorations, no preapical lesions were observed in all groups, which proved that all these materials were biocompatible to the dentin-pulp organ (Figure 2).

In the current study, as shown in Figures 3, 4, calcium hydroxide recorded higher scores of POH at one month, three months, and six months than TheraCal LC and bioactive adhesives, but no difference was found immediately after placement and at one-month intervals. These results may be due to the properties and characteristics of the calcium hydroxide material, which has a high pH once placed, potent antibacterial properties and favorable reparative dentin formation, and low-grade initial irritation to the pulp with precipitation of calcium salts that initiate dentin bridge formation [37]. However, by time, as reported by literature and previous studies, this material suffers from gradual degradation over time as it does not adhere to the dentin with the formation of tunnel defects within the dentin bridge. This results in the decrease of sealing capability and leads to microleakage and increased number of inflammatory cells and pulpal inflammation. All of this result in failure to provide a barrier under the resin composite restoration, which may lead to penetration of the residual monomers to the pulp evolving pain and sensitivity. This explanation was proven by the results of this study, which recorded a higher score of hypersensitivity in calcium hydroxide after six months than the scores recorded in the preceding time periods [19,38].

As shown in Figures 3, 6, universal adhesives recorded higher scores of hypersensitivity at three months and six months when placed under resin composite than that of TheraCal LC and bioactive adhesives. In addition, significant scores of hypersensitivity recorded were higher than the preceding time periods. This was in agreement with other studies that explained this on the basis that unconverted monomers that remain in the outer layer of adhesive that is in contact with oxygen may escape and diffuse through dentinal tubules and pulp, causing hypersensitivity [39,40]. In addition, All-Bond Universal used in this study has BIS-GMA in its composition, which may produce undesirable effects as it is easily solubilized by solvents, such as ethanol, present in the primer of this adhesive. Also, HEMA and water present in this adhesive may cause pulp reactions due to its solubility in aqueous Solutions owing to its hydrophilicity and can form water permeable adhesive layer, thus compromising bond by time and causing hypersensitivity [41].

As observed in Figures 6, 7, TheraCal LC yielded a low score of hypersensitivity compared to others when placed under resin composite or glass ionomer at different time intervals. These results coincide with other results that confirmed the bioactivity of TheraCal LC and its ability to form dentin bridges. Furthermore, TheraCal LC has the ability to cause a rise in pH to 10-11, which promotes pulp healing and decreases hypersensitivity. This PH resumes its normality after a few weeks [41,42]. Moreover, other studies confirm the bioactivity of TheraCal LC and its ability to form reparative dentin, which was effective as calcium hydroxide despite its composition of resin and silanol, but the material has the capability of precipitation of calcium phosphate deposits. TheraCal LC has the ability to enhance sealing ability by chemical binding to the dentin, allowing the release of calcium phosphate ions, excellent biological properties, and lower solubility over time, which aids in decreasing hypersensitivity [43,44].

Bioactive self-etch adhesive FL-Bond II showed the lowest score of hypersensitivity in comparison to other groups under both resin composite and glass ionomer at all time periods (Figures 3, 6). These results were in agreement with previous studies that reported low frequency of POH for the self-etch adhesives for its ability to dissolve the smear layer and integrate as a part of the hybrid layer and promote a reliable adhesion junction [14]. Furthermore, other studies relied on using FL-Bond II in decreasing hypersensitivity by producing a significant change to the dentin by increasing the resistance to secondary caries and enhancing its sealing capability. This is due to its composition of S-PRG surface pre-reacted glass particles, which have six different ions in its composition (calcium, silicon, boron, sodium, strontium, and fluoride). They were found to be released from this adhesive material and promote dentin remineralization, increase resistance to secondary caries, and decreasing hypersensitivity. Moreover, this material has higher filler loading, which provides excellent sealing capability [45]. Other studies focused on the potentiality of self-adhesives in decreasing hypersensitivity. The second bottle applied was hydrophobic monomers containing water and solvent-free resins, which were applied to the primer-treated dentin [27].

There was no statistically significant difference between resin composite and glass ionomer restorations with the different lining materials in different time periods (Figures 5, 6). This can be explained by the fact that even a thin layer of dentin bridge when doing indirect pulp lining is quite enough to protect pulp against both the restorative material and the strategy and technique of adhesive placement so that different restorative materials will not have a significant effect regarding POH [15,46].

Despite the sample size being relatively small in this in vivo study, the results are more evident and useful than if the study had been conducted in vitro. The patients reported some difficulties in reporting the type of pain, whether mild or moderate, when using a verbal analog scale. This was when the air syringe or ice stick was applied. Yet, making a quantitative description of pain has been more accurate than a qualitative one [33].

Although this investigation was conducted for a six-month period due to the availability of patients for follow-up, it gives precise results and data that were sufficient for measuring and recording POH. Also, a long clinical trial would offer a broader and more precise results [37].

POH may also be caused by the penetration of the components of adhesives into pulp by micro or nano leakage, allowing the movement of fluids inside dentinal tubules in accordance with hydrodynamic theory by Brännström. Also, different factors can provoke sensitivity, such as polymerization shrinkage of resin composite, adhesive strategy technique regarding its placement inside the cavity, intensity of light curing unit, cuspal deflection, and different adhesive restorations [11].

Study limitations

The limitation of this study is that it was conducted by an experienced operative dentist, and it may not be applicable to a general dental practice situation. Furthermore, the thickness of the lining material may also affect the results of POH. Therefore, it should be further investigated whether a thin or thick lining will positively or negatively affect the results.

Clinical recommendations

Based on this clinical study, it is advisable to use TheraCal LC and bioactive adhesives in class II deep cavity preparations if the indirect pulp lining technique is adopted. Follow-up of the patients is highly recommended if planning calcium hydroxide or universal hydrophilic adhesives to observe the effect of these materials on causing hypersensitivity after a period of time. Other studies should be conducted on different patients' dentitions for different classes to obtain more precise and accurate evidence regarding the POH.

## Conclusions

Based on the results of this clinical study, it can be concluded that using different lining materials, such as indirect pulp lining in deep cavities, may promote POH. TheraCal LC and bioactive adhesives are considered promising materials for indirect pulp lining in deep cavities. Sealing of the cavity with resin composite or glass ionomer restorations was not a dependent factor regarding POH. Also, it seems that time is a crucial factor for investigating the effect of different lining materials on hypersensitivity.

POH might be brought out by the correlation between multiple factors such as restoration technique, the clinical condition of the tooth, remaining dentin bridge thickness, the restorative material, shape, size, and extension of cavity preparation, and the use of pulp lining materials. Therefore, unpredictable POH may possibly occur.
